# Comparison of Brain Oxygen Metabolic Parameters Between Constrained qBOLD and Whole‐Brain Oximetric Methods at Baseline and in Response to a Physiologic Stimulus

**DOI:** 10.1002/nbm.70120

**Published:** 2025-08-11

**Authors:** Kathryn M. Jaroszynski, Hyunyeol Lee, Michael C. Langham, Felix W. Wehrli

**Affiliations:** ^1^ Department of Radiology Perelman School of Medicine, University of Pennsylvania Philadelphia Pennsylvania USA; ^2^ Department of Bioengineering, School of Engineering and Applied Sciences University of Pennsylvania Philadelphia Pennsylvania USA; ^3^ School of Electronic and Electrical Engineering Kyungpook National University Daegu Republic of Korea

**Keywords:** 3D MRI, blood flow, brain mapping, brain metabolism, oxygenation

## Abstract

The measurement of cerebral oxygen metabolism is important to understand and treat many disorders. Constrained quantitative BOLD (qBOLD) MRI is a calibration‐free method for 3D voxel‐wise whole‐brain mapping of brain oxygen metabolism. This study aimed to evaluate the agreement between constrained qBOLD and global oximetry methods both at baseline and in response to a caffeine stimulus. Healthy volunteers (*N* = 10, age 30 ± 8 years) were imaged with constrained qBOLD, MOTIVE (metabolism of oxygen via T_2_ and interleaved velocity encoding), dual‐slice (DS), and single‐slice (SS) OxFlow. Subjects were then given a 200 mg caffeine pill and imaged at 2‐s temporal resolution immediately thereafter for 30 min by SS‐OxFlow. After 30 min, the baseline protocol was repeated. Constrained qBOLD uses prior constraints to the QSM + qBOLD model to solve for voxel‐wise oxygen extraction fraction (OEF). Quantification of cerebral blood flow (CBF) was accomplished for qBOLD from a separate measurement via pseudo‐continuous arterial spin labeling (pCASL) to yield CMRO_2_. Constrained qBOLD measured OEF (31 ± 5% gray matter [GM], 31 ± 6% white matter [WM] at baseline; 36 ± 7 GM, 35 ± 8 WM post‐caffeine) was in good agreement with global oximetry methods DS‐OxFlow (30 ± 4, 37 ± 5), SS‐OxFlow (31 ± 4, 37 ± 4), and MOTIVE (32 ± 5, 39 ± 5). Temporal data showed a gradual increase in OEF with a commensurate reduction in CBF while the caffeine was taking effect. No significant change in CMRO_2_ was noted with any of the techniques. Regional analysis of the basal ganglia, hippocampus, and thalamus found there was a significant increase in OEF post caffeine. The results indicate constrained qBOLD to yield OEF with negligible bias to global oximetry methods, both at baseline and post caffeine. The results also suggest that constrained qBOLD has the sensitivity to detect changes in oxygen metabolism due to a vasoconstrictive stimulus.

## Graphical abstract

This study aimed to evaluate the agreement between constrained qBOLD and global oximetry methods both at baseline and in response to a caffeine stimulus. The results indicate constrained qBOLD to yield OEF with negligible bias to global oximetry methods, both at baseline and post caffeine. The results also suggest that constrained qBOLD has the sensitivity to detect changes in oxygen metabolism due to a vasoconstrictive stimulus.

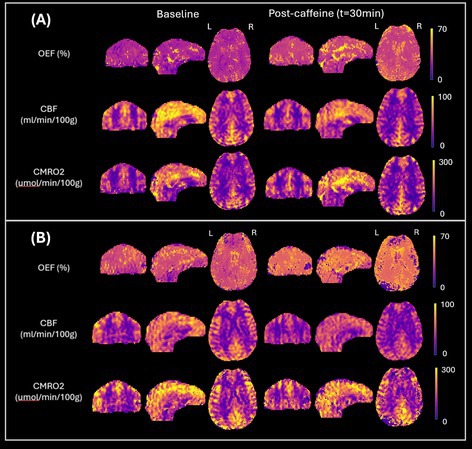

**Abbreviations**
AUSFIDE: alternating “unbalanced steady‐state free‐precession FID and echo”
CBF: cerebral blood flow
CBV_v_
: venous cerebral blood volume
CMRO_2_
: cerebral metabolic rate of oxygen consumption
MOTIVE: metabolism of oxygen via T_2_ and interleaved velocity encoding
OEF: oxygen extraction fraction
PC: phase contrast
pCASL: pseudo‐continuous arterial spin labeling
qBOLD: quantitative blood oxygen level dependent
S_v_O_2_
: venous oxygen saturation
VS‐VSL: velocity‐selective venous spin labeling

## 1 Introduction

The understanding of brain oxygen metabolism is important for the study and treatment of many disorders, including obstructive sleep apnea [1], steno‐occlusive disease [2], brain tumors [3], and neurodegenerative disease [4]. While comprising only 2% of adult body weight, the human brain accounts for 20% of the body's total energy requirements, making it prone to malfunction in the event of compromised supply of oxygen [5]. Imaging techniques, including positron emission tomography (PET) and magnetic resonance imaging (MRI), are currently used as methods for monitoring brain oxygen metabolism for investigating the various cerebral malfunctions.

While the clinical standard for assessment of cerebral oxygen metabolism is ^15^O PET, this method is costly, complex, time consuming, and exposes patients to ionizing radiation [6]. Several MRI‐based oximetry methods have made inroads since their inception to assess both global [7, 8, 9, 10, 11, 12] and regional [13, 14, 15, 16, 17, 18] oxygen metabolism, providing a noninvasive, ionizing radiation‐free alternative to PET imaging. In MRI, the quantitative assessment of oxygen metabolism in organs makes use of the paramagnetic properties of deoxyhemoglobin, either through quantification of T_2_ (transverse relaxation time) of blood water in a draining vein [8, 10, 11, 12, 19], relative magnetic susceptibility between whole blood and adjacent tissue [9, 20], or by analyzing the signal decay in the extravascular tissue that is due to the induced magnetic field [18].

Whole‐organ (global‐scale) MRI oximetry methods include TRUST (T_2_‐relaxation‐under‐spin‐tagging) [19], MOTIVE (metabolism of oxygen via T_2_ and interleaved velocity encoding) [8], and OxFlow [9]; the latter two methods quantify venous oxygen saturation (S_v_O_2_) and cerebral blood flow (CBF) in a single pass. However, these techniques quantify only global‐scale CMRO_2_ and therefore do not allow investigation of specific brain regions. Calibrated BOLD (cBOLD) permits voxel‐wise (local‐scale) estimation of CMRO_2_; however, this method requires knowledge of a calibration constant that must be determined from an initial hypercapnic or hyperoxic gas breathing challenge [17, 21, 22, 23].

Alternatively, quantitative BOLD (qBOLD) methods, based on the Yablonskiy model for R_2_′ decay [15, 18], provide a calibration‐free local‐scale estimation of oxygen extraction fraction (OEF). This method is built on the principle that, under some conditions, the radiofrequency (RF) reversible portion of the transverse relaxation rate (R_2_′) is linearly proportional to S_v_O_2_ and deoxygenated blood volume (DBV) [15, 16, 24]. However, conventional methods such as GESSE [25] and GESFIDE [26] are limited to 2D quantification because of impractically long 3D scan times. Also limiting is the mutual coupling of S_v_O_2_ and DBV, rendering the model error prone, and their failure to account for non‐heme brain iron [13, 27]. Quantitative susceptibility mapping (QSM) has been shown to provide comparable OEF results to cBOLD; however, two physiological states were needed to separate non‐heme iron contributions [28]. QSM combined with qBOLD has more recently been shown to achieve rapid 3D CMRO_2_ mapping while accounting for non‐blood tissue susceptibility [13, 29, 30, 31]. However, the method does not directly quantify R_2_′ or cerebral blood volume (CBV) and instead relies on cluster analysis or deep learning algorithms to reduce noise in the model [14, 32, 33].

Some of the present authors had previously developed a method termed “constrained qBOLD,” an approach that is based on a separate measurement of R_2_′ and CBV as additional prior constraints to the QSM + qBOLD model [34, 35, 36], which reduces noise and improves accuracy of quantification by decoupling S_v_O_2_ and DBV. While constrained qBOLD has been validated for repeatability and sensitivity to hypercapnic gas stimuli [37], it has not been compared with some of the global oximetry methods described above. Relative agreement between methods is particularly important when the ground truth is not known, which is often the case in MRI oximetry [37, 38, 39, 40, 41]. This paper aims to compare constrained qBOLD with T_2_‐based (MOTIVE) [8] and susceptometry‐based (OxFlow) global oximetry methods, both at baseline and in response to a physiological stimulus in the form of caffeine supplementation.

## 2 Experimental

### 2.1 Physical and Technical Principles

In the present study, global and local scale methods were used to quantify cerebral metabolic parameters including OEF, CBF, and CMRO_2_ at baseline and in response to caffeine, a vasoconstrictive stimulus.

#### 2.1.1 CMRO_2_ Quantification

To quantify CMRO_2_, MR oximetry methods invoke Fick's principle [10]:

(1) $${\text{CMRO}}_{2}=\mathrm{CBF}\left(S_{a}O_{2}-S_{v}O_{2}\right)\cdot C_{a}$$

In Equation (1), *C*_*a*_ is the oxygen carrying ability of arterial blood, which is given by the oxygen binding capacity of red blood cells (C_rbc_ = 19.93 μmolO_2_/mL) multiplied by hematocrit. S_a_O_2_ is assumed to be 98%, whereas hematocrit was determined individually for each subject by a finger prick test (Hemocue, Sweden). Brain mass was calculated by multiplying the brain volume estimation using an MPRAGE by the density of brain tissue, assumed to be 1.05 g/cm^3^. S_v_O_2_ was determined from the MR technique, whether global (MOTIVE, OxFlow) or voxel‐wise (qBOLD).

#### 2.1.2 Global Scale Methods

OxFlow simultaneously measures S_v_O_2_ in the superior sagittal sinus (SSS) with a susceptometry‐based method, and average CBF via phase contrast (PC) imaging at the neck. Susceptometry‐based oximetry methods exploit the intrinsic susceptibility difference in venous whole blood relative to surrounding tissue (see Jain et al. [9] for full details of the dual‐slice [DS] OxFlow sequence). In brief, this sequence, referred to in this work as DS OxFlow, uses an acquisition scheme that toggles between two axial data acquisition schemes at the base of the skull for flow encoding of the blood in the feeding arteries (internal carotid and vertebral arteries) and field mapping at the level of the SSS, from which S_v_O_2_ is obtained.

The second OxFlow sequence evaluated here, termed single‐slice (SS) OxFlow, in contrast, was designed for temporally resolved imaging. This is achieved by data collection at the SSS only, for both blood flow velocity and S_v_O_2_ quantification. In its original implementation [20], high temporal resolution of 2 s was achieved using a view‐sharing approach. Because the SSS drains only about 50% of the brain, the blood flow rate measured at this site needs to be scaled up, which was done in a manner analogous to that described by Rodgers et al. [20]. The greater efficiency of the spiral sampling scheme obviates the need for view sharing to achieve a true temporal resolution of 2 s.

For both DS and SS OxFlow, ROIs were drawn manually to quantify the average relative phase difference between the SSS and the surrounding brain parenchyma on the field map for calculation of S_v_O_2_ [9], which was computed from Equation (2):

(2) $$S_{v}O_{2}=\left[1-\frac{2\left|∆\phi \right|}{\gamma ∆\chi_{\mathrm{do}}B_{0}\Delta \mathrm{TE}\left(\;\cos^{2}\theta -\frac{1}{3}\right)\mathrm{Hct}}\right]$$

where ∆ϕ is the average phase difference between the SSS and surrounding tissue, γ is the gyromagnetic ratio for protons in water (42.58 MHz/T), $∆\chi_{\mathrm{do}}$ is the susceptibility difference between fully deoxygenated and fully oxygenated red blood cells (4π · 0.27 ppm), θ is the vessel tilt angle with the respect to the main field (B_0_), ΔTE is the interecho spacing between two equal‐polarity gradient‐recalled echoes, and Hct is the volume fraction of erythrocytes in whole blood.

**FIGURE 1:**
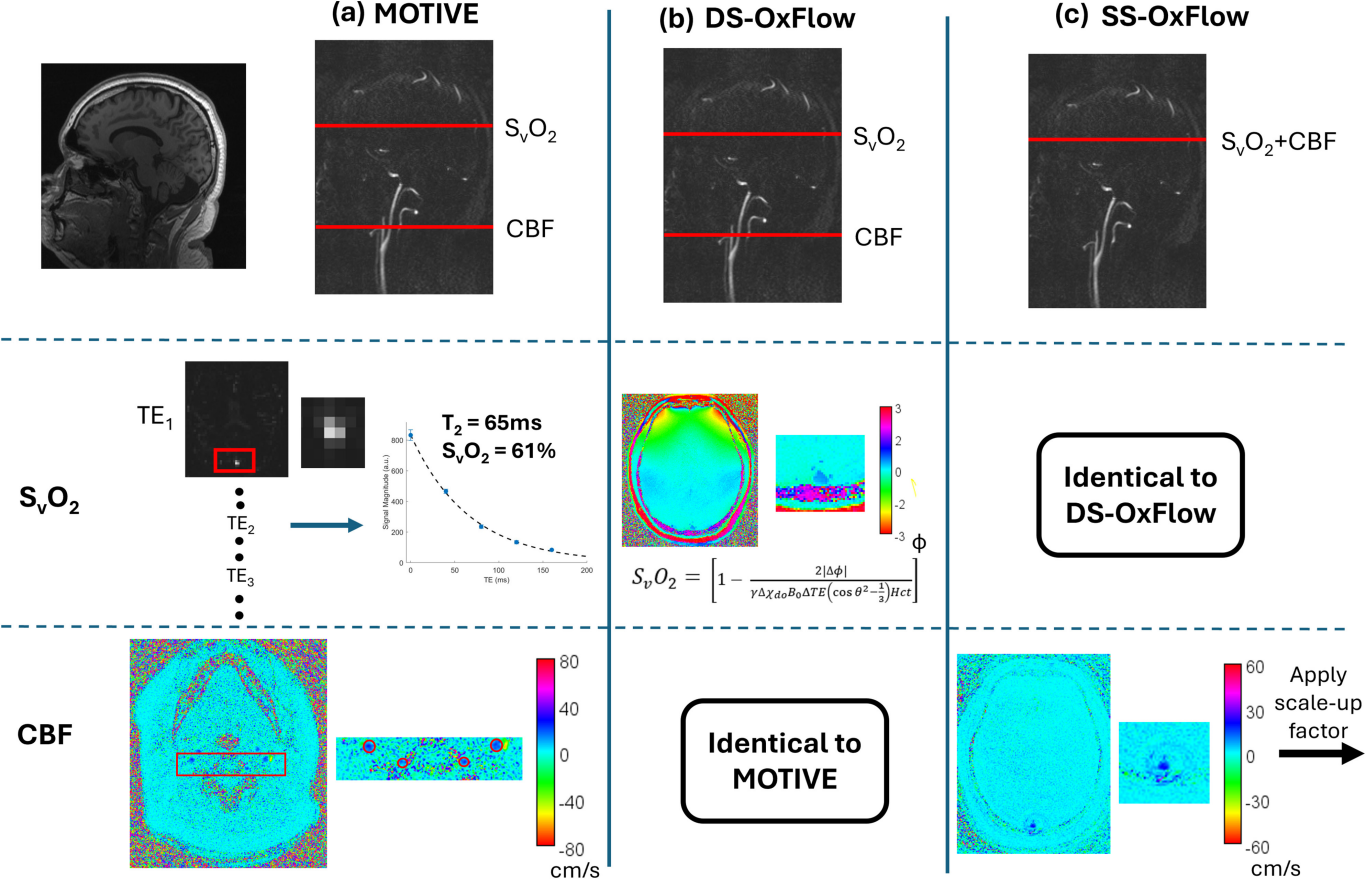
Overview of global techniques: (a) MOTIVE, (b) DS‐OxFlow, and (c) SS‐OxFlow. MOTIVE and DS‐OxFlow measure cerebral blood flow at the neck using phase contrast imaging. SS‐OxFlow measures the blood flow in the superior sagittal sinus (SSS) and scales this value to represent total blood flow rate. Both OxFlow sequences exploit the susceptibility difference between the blood in the SSS and the surrounding tissue to calculate S_v_O_2_. MOTIVE, in contrast, measures T_2_ and converts the value to S_v_O_2_ based on a calibration curve.

The third whole‐organ MRI oximetry method used, MOTIVE, measures S_v_O_2_ at the SSS, along with the average CBF rate of the feeding arteries at the neck. Unlike OxFlow, MOTIVE makes use of the O_2_ saturation dependence of blood water proton transverse relaxation time T_2_ [42] analogous to TRUST [19], except that the method is non‐subtractive, using instead soft‐tissue suppression to isolate the vessels of interest [8]. In MOTIVE, T_2_ is quantified and converted to S_v_O_2_ via a calibration curve. Figure 1 describes each global method, and a table describing all three global sequences can be found in Table S1.

#### 2.1.3 Local Scale Method (Constrained qBOLD)

The constrained qBOLD approach utilizes two custom pulse sequences to quantify spatially resolved 3D maps of R_2_, R_2_′, relative magnetic susceptibility (∆χ), macroscopic field inhomogeneity (∆B_0_), as well as venous CBV (CBV_v_) [34, 35, 36]. The first sequence, termed alternating “unbalanced steady‐state free‐precession FID and echo” (AUSFIDE), consists of two unbalanced steady‐state‐free‐precession (SSFP) components (SSFP‐FID and SSFP‐ECHO) to achieve 3D encoding of R_2_ and R_2_′ [35]. Within each SSFP mode, a group of gradient‐recalled signals are sampled to obtain temporal signal decays, with rate constants of R_2_ + R_2_′ (=R_2_*) and R_2_ − R_2_′ for FID and ECHO, respectively. In addition to R_2_ and R_2_′ estimation from magnitude processing, ∆B_0_ and ∆χ are obtained through processing of the phase data [35]. A full description of this sequence can be found in reference [35].

The second sequence, termed “velocity‐selective venous spin labeling” (VS‐VSL), isolates the venous blood signal to obtain a measurement of venous blood volume (CBV_v_). The sequence begins with a slab‐selective saturation and spatially nonselective inversion recovery, designed to suppress the signal from arterial blood and cerebral spinal fluid (CSF) [34]. A velocity‐selective pulse train follows, consisting of control/tag images that are subtracted to eliminate static tissue signal and isolate venous blood using 3D turbo spin‐echo readout. A simplified VS‐VSL signal model yields CBV_v_ parametric map directly from the control/tag difference [34].

Constrained qBOLD (henceforth referred to simply as “qBOLD”) takes in the preliminary parameters estimated from AUSFIDE (R_2_, R_2_′, ∆χ, ∆B_0_) and VS‐VSL (CBV_v_) as prior information for solving the following nonlinear inverse problem [36]:

(3) $$\text{argmin}\left(\Theta \right)\sum_{\mathrm{TE}}{\left|y\left(\mathrm{TE}\right)-\Xi \left(\Theta ,\mathrm{TE}\right)\right|}^{2}+w{\left|∆\chi -\Psi \left(\Theta \right)\right|}^{2}+p{\left|R_{2}^{\prime }-Y\left(\Theta \right)\right|}^{2}$$

**FIGURE 2:**
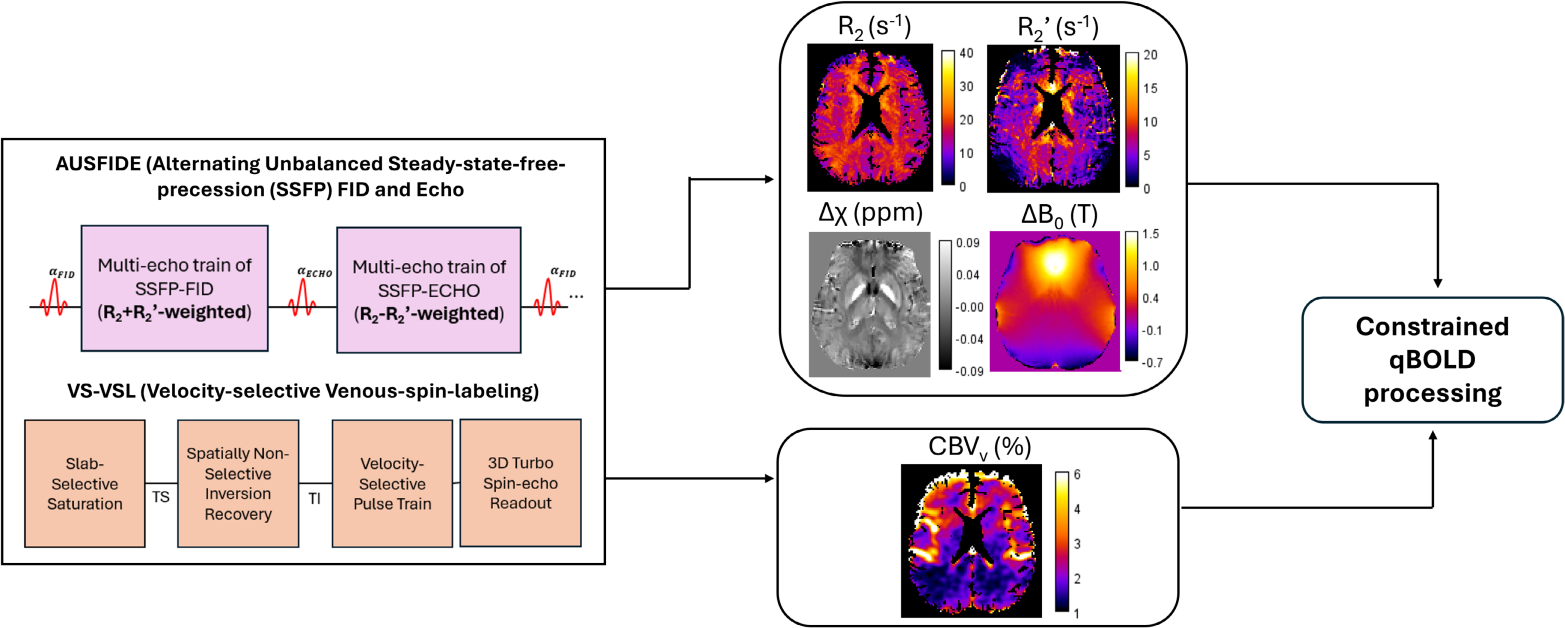
Overview of qBOLD protocol. Alternating unbalanced steady‐state‐free‐precession FID and ECHO (AUSFIDE) returns estimates of R_2_, R_2_′, magnetic field inhomogeneity (ΔB_0_), and voxel susceptibility (Δχ). Velocity‐selective venous‐spin‐labeling (VS‐VSL) yields estimates of venous cerebral blood volume (CBV_v_). The estimated parameters then serve as input (prior constraints) to the nonlinear qBOLD model (Equation 3).

In Equation (3), y is the vectorized AUSFIDE signal at echo time TE. The term Ξ is the corresponding model that represents the temporal signal decay with rate constants of R_2_ and R_2,nh_′ (contributions from non‐heme iron). The quantity Θ describes the set of parameters being solved for, which includes DBV, R_2,nh_′, non‐blood voxel susceptibility (∆χ_nb_), and S_v_O_2_. Ψ accounts for the four pools of voxel susceptibilities, comprising deoxygenated arterial and venous blood, fully oxygenated blood and non‐blood tissue. Y decomposes R_2_′ into heme and non‐heme iron contributions. Lastly, w and p are regularization parameters. CBV_v_ is used to guide the solution of DBV. Full details can be found in reference [36]. This optimization problem is solved on a voxel‐by‐voxel basis. An overview of the qBOLD protocol is given in Figure 2. In addition to AUSFIDE and VS‐VSL for S_v_O_2_ quantification, a pseudo‐continuous arterial spin labeling (pCASL) sequence [43] was run to measure voxel‐wise CBF in mL/min/100 g.

### 2.2 Data Acquisition and Analysis

All imaging was conducted on a 3T Prisma MRI scanner (Siemens, Germany) with a 64‐channel head coil. Unless otherwise noted, all image processing and data analysis was done using MATLAB.

#### 2.2.1 Experimental Protocol

Eleven subjects (mean age 30 ± 8 years, eight male) were recruited to participate, yielding both whole‐brain and regional qBOLD data in a single MRI session each. Subjects were asked to refrain from caffeine consumption from 8:00 p.m. the night before. An overview of participant demographics is given in Table S2. Hematocrit was determined from a finger prick test as described in Section 2.1.1. The protocol was approved by the Institutional Review Board of the University of Pennsylvania. Informed consent was obtained for all elements of the study, in accordance with IRB requirements. The MRI protocol began with approximately 30 min of baseline scanning, including the qBOLD protocol (AUSFIDE, VS‐VSL, pCASL), and acquisition of data with the three global methods: DS‐OxFlow, SS‐OxFlow, and MOTIVE. Global data was acquired first, with a combined total scan time of approximately 5 min, including localization scans and slice selection. One subject's data was omitted due to subject motion corruption, which precluded data interpretation; therefore, reducing the analysis to data from 10 subjects.

AUSFIDE parameters were as follows: repetition time (TR)=30ms, FOV = 240 × 240 × 120 mm^3^, matrix size = 160 × 160 × 40, number of echoes = 17, first TE (FID) = 1.6 ms, first TE (ECHO) = 2.2 ms, echo spacing = 1.5 ms. With a slice oversampling rate of 25%, yielding a scan time of 8 min. The parameters for VS‐VSL were TR = 3 s, FOV = 240 × 240 × 180 mm^3^, matrix size = 72 × 72 × 60, saturation time = 1.65 s and inversion time = 1.14 s for a total scan time of 3.3 min. Lastly, a 3D pseudo‐continuous ASL sequence with a stack‐of‐spirals readout scheme [43] was run with the following parameters: FOV = 240 × 240 × 140 mm^3^, matrix size = 68 × 68 × 40, post‐labeling delay = 2 s, with 6 control/tag pairs and an acquired scan time of 4.3 min. For brain volume estimations and registration purposes, T1‐weighted MPRAGE images were also collected at 1 mm^3^ voxel size. The entire qBOLD/pCASL protocol was approximately 20 min long.

#### 2.2.2 Caffeine Challenge

After completion of baseline scans, the subject was removed from the scanner and allowed to exit the room. Once in the control room and after a few minutes of rest, the participant was given a 200 mg caffeine pill (Nutricost, UT) and escorted back into the MRI room for repeat scanning. Subjects were scanned for 30 min of time‐resolved imaging (2 s temporal resolution) via SS‐OxFlow while the caffeine was taking effect to gain insight into the time course of the physiologic response. After the 30 min of continuous scanning with SS‐OxFlow, all baseline imaging was repeated. The total procedure time (baseline and caffeine) was 1 h and 30 min. One subject was unable to tolerate the 1‐h second scan, so no temporal data could be collected.

#### 2.2.3 Data Analysis

For comparison with global methods, all voxels of the qBOLD brain maps (excluding CSF spaces) were used to compute a global average. For regional comparisons, white and gray matter (GM) were automatically segmented from the T1‐MPRAGE image using the SPM12 software [44]. Further, specific brain regions were isolated with Freesurfer [45]. MPRAGE images, along with all segmentations, were registered to the first echo of the FID in AUSFIDE, from which regional CBF, CMRO_2_, and OEF were determined.

For inter‐method comparison of key parameters, box plots were created for OEF, CBF, and CMRO_2_ both pre‐ and post‐caffeine, and one‐way ANOVA (*α* = 0.05) was conducted for both states. Pearson correlation and Bland–Altman analysis were also conducted for comparing qBOLD with each global method. To evaluate each method's sensitivity to the caffeine stimulus, *p*‐values for paired *t*‐tests (*α* = 0.05) conducted on pre‐ and post‐caffeine data for all global methods as well as constrained qBOLD were calculated. In addition to a whole‐brain average, pre‐post regional effects in cerebral oxygen metabolism were investigated from the qBOLD data, including basal ganglia, hippocampus, thalamus, precentral gyrus, and GM and white matter (WM). Lastly, box plots were compared for whole‐brain, GM, and WM qBOLD before and after caffeine stimulation. The temporally resolved data collected while the caffeine was taking effect was analyzed for CBF, OEF, and CMRO_2_.

## 3 Results

### 3.1 Comparison of Cerebrovascular‐Metabolic Parameters Between Constrained qBOLD and Whole‐Brain Oximetry Methods

The average baseline and post‐caffeine values of OEF, CBF, and CMRO_2_ for all 10 subjects and each technique are given in Table 1. The mean values of OEF in percent were 30 ± 4, 31 ± 4, 32 ± 5, and 31 ± 5 at baseline and 37 ± 5, 37 ± 4, 39 ± 5, and 35 ± 7 post‐caffeine for DS‐OxFlow, SS‐OxFlow, MOTIVE, and qBOLD, respectively. Individual T_2_ and S_v_O_2_ values are also shown in Table 2. Importantly, there were no significant differences in the quantified vascular‐metabolic parameters between techniques. Mean CBF values of 50 ± 9, 52 ± 9, 51 ± 9, and 55 ± 19 at baseline and 40 ± 8, 45 ± 8, 40 ± 7, and 47 ± 14 mL/min/100 g post‐caffeine were found for DS‐OxFlow, SS‐OxFlow, MOTIVE, and qBOLD, respectively. There was no significant mutual bias between the four methods. The inter‐method mean differences were greater for CMRO_2_: 118 ± 15, 128 ± 18, 130 ± 16, and 144 ± 27 (baseline) and 115 ± 17, 131 ± 25, 123 ± 19, and 139 ± 34 (post‐caffeine) μmol/min/100 g for the four methods. Further, baseline one‐way ANOVA was significant, and post hoc *t*‐tests showed CMRO_2_ averages for DS‐OxFlow to differ somewhat from those obtained by qBOLD (118 versus 144 μmol/min/100 g, *p* = 0.03). On the other hand, post‐caffeine CMRO_2_ did not differ among techniques (*p* = 0.18).

**TABLE 1:** Group‐averages of OEF, CBF, and CMRO_2_ in 10 subjects studied at baseline (pre) and in response to a caffeine challenge (post). *p*‐values are shown for each technique pre vs. post caffeine as well as for one‐way ANOVA comparing all techniques.

	OEF (%)			CBF (mL/min/100 g)			CMRO_2_ (μmol/min/100 g)		
	Pre	Post	*p*‐value^b^	Pre	Post	*p*‐value^b^	Pre	Post	*p*‐value^b^
DS OxFlow	30 ± 4	37 ± 5	0.002**	50 ± 9	40 ± 8	< 0.001**	118 ± 15	115 ± 17	0.54
SS OxFlow	31 ± 4	37 ± 4	0.002**	52 ± 9	45 ± 8	0.005**	128 ± 18	131 ± 25	0.64
MOTIVE	32 ± 5	39 ± 5	0.001**	51 ± 9	40 ± 7	< 0.001**	130 ± 16	123 ± 19	0.16
qBOLD^a^	31 ± 5	35 ± 7	0.01**	55 ± 19	47 ± 14	0.06	144 ± 27	139 ± 34	0.47
*p*‐value^c^	0.73	0.61		0.79	0.33		0.05*	0.18	

Abbreviations: CBF, cerebral blood flow; CMRO_2_, cerebral metabolic rate of oxygen consumption; OEF, oxygen extraction fraction.

^a^ All voxels of the qBOLD maps were averaged together for comparison.

^b^ *p*‐values for post hoc comparisons pre/post caffeine.

^c^ *p*‐values from one‐way ANOVA analysis (*α* = 0.05).

* Significant *p*‐value from one‐way ANOVA analysis (*α* = 0.05).

** Significant *p*‐value from paired *t*‐test (*α* = 0.05).

**TABLE 2:** Hematocrit, T_2_, and S_v_O_2_ values for each subject studied.

Subject	Hct (fraction)	T_2_ (ms)^a^		S_v_O_2_ (%)							
				DS Oxflow		SS Oxflow		MOTIVE		qBOLD	
		Pre	Post	Pre	Post	Pre	Post	Pre	Post	Pre	Post
1	0.321	88	65	75	68	75	68	74	64	75	70
2	0.360	80	65	63	59	63	62	66	59	61	60
3	0.435	62	64	66	64	65	63	62	62	62	57
4	0.417	66	63	69	68	70	69	63	62	71	73
5	0.40	66	53	65	58	67	59	62	55	67	59
6	0.424	80	67	68	62	65	60	70	64	68	65
7	0.388	66	53	67	54	67	58	62	54	70	62
8	0.45	73	54	72	58	69	57	68	58	62	51
9	0.479	80	77	70	68	69	67	72	71	67	68
10	0.450	69	51	72	66	69	62	66	55	75	72

^a^ T_2_ values calculated from MOTIVE sequence using the calibration curve by Lu et al. [46]. See Section 2.1.2 of methods for more details. Reported T_2_ values are an average over three repetitions.

**FIGURE 3:**
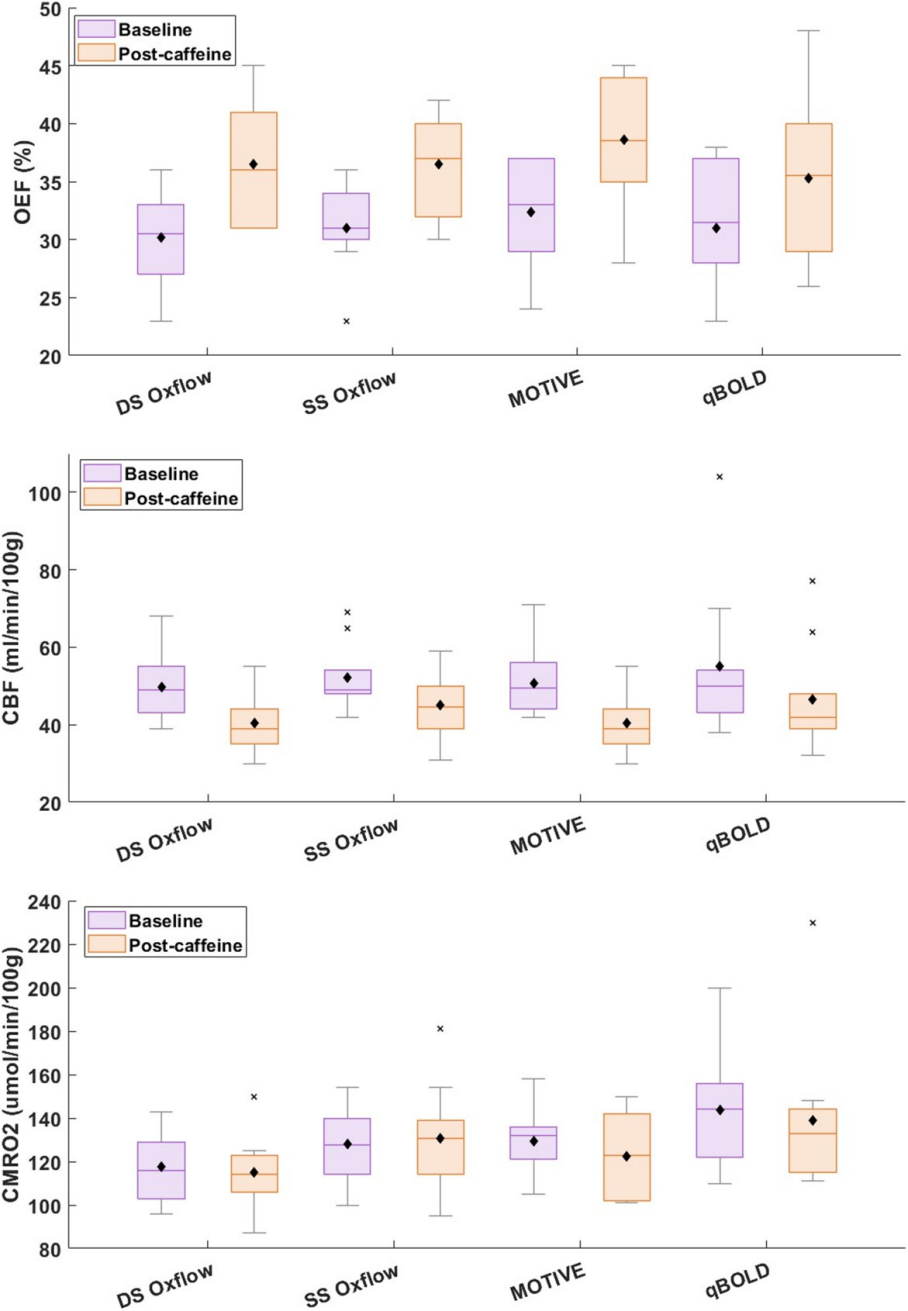
Baseline and post‐caffeine values for OEF, CBF, and CMRO_2_ from *N* = 10 study subjects. Means are depicted in black. There is little difference in measurement between techniques both pre‐ and post‐caffeine. Post hoc *p*‐values for pre/post caffeine post hoc analysis were 0.002, 0.002, 0.001, and 0.01 for DS‐OxFlow, SS‐OxFlow, MOTIVE, and qBOLD measured OEF, respectively; < 0.001, 0.005, < 0.001, and 0.06 for CBF, and 0.54, 0.64, 0.16, and 0.47 for CMRO_2_.

Boxplots are shown for OEF, CBF, and CMRO_2_ for both metabolic states in Figure 3. Importantly, the group values of OEF and CBF were found to be very similar across techniques, with somewhat greater variability in CMRO_2_, given error propagation resulting from the multiplication of the two measured quantities (arteriovenous difference and CBF).

**FIGURE 4:**
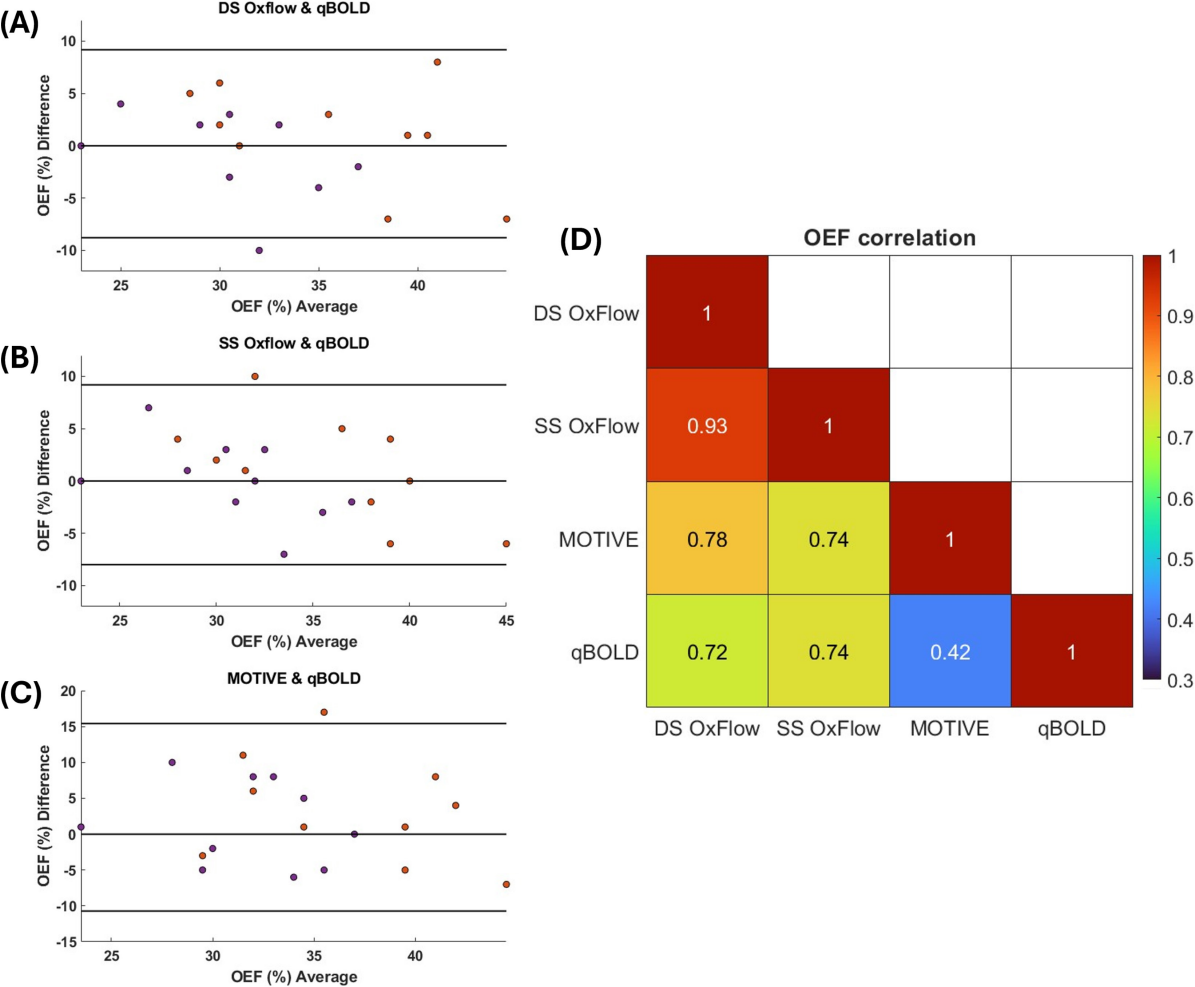
Relative agreement between qBOLD and the whole‐brain techniques for OEF at baseline and post‐caffeine challenge. (A) DS‐OxFlow, (B) SS‐OxFlow, (C) MOTIVE. The Pearson correlation coefficients are shown in panel (D).

Associations and mutual bias between qBOLD and global measurements for OEF are shown in Figure 4. OxFlow displayed the highest level of agreement. Further, mutual correlations indicate that the qBOLD derived parameters generally tracked those from whole‐brain techniques well (*R* = 0.72 for DS‐OxFlow, and *R* = 0.74 for SS‐OxFlow, all *p* ≤ 0.005). Correlation was weaker between qBOLD and MOTIVE (*R* = 0.42, *p* = 0.06), consistent with lower mutual agreement. Correlation matrices for CBF and CMRO_2_ are shown in Figure S1. There was moderate correlation between pCASL derived CBF (qBOLD) and global methods, but CMRO_2_ was only weakly correlated.

### 3.2 Effect of Caffeine Challenge

**FIGURE 5:**
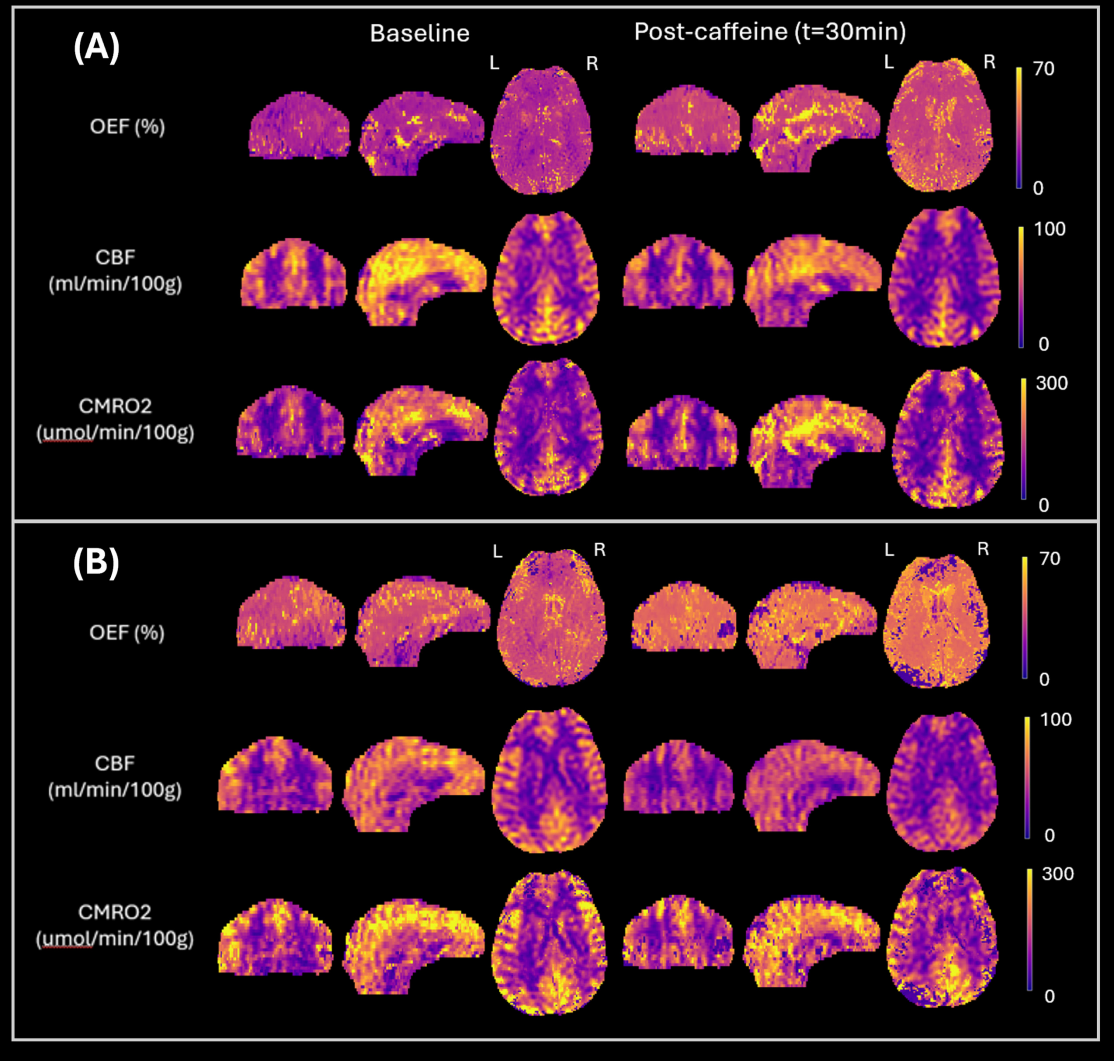
Baseline and post‐caffeine OEF, CBF, and CMRO_2_ maps from two subjects: (a) 33‐year‐old female, (b) 24‐year‐old male. Both subjects display the expected reduction in cerebral blood flow and compensatory increase in oxygen extraction fraction but exhibit minimal change in CMRO_2_ in accordance with the isometabolic nature of the stimulus.

**FIGURE 6:**
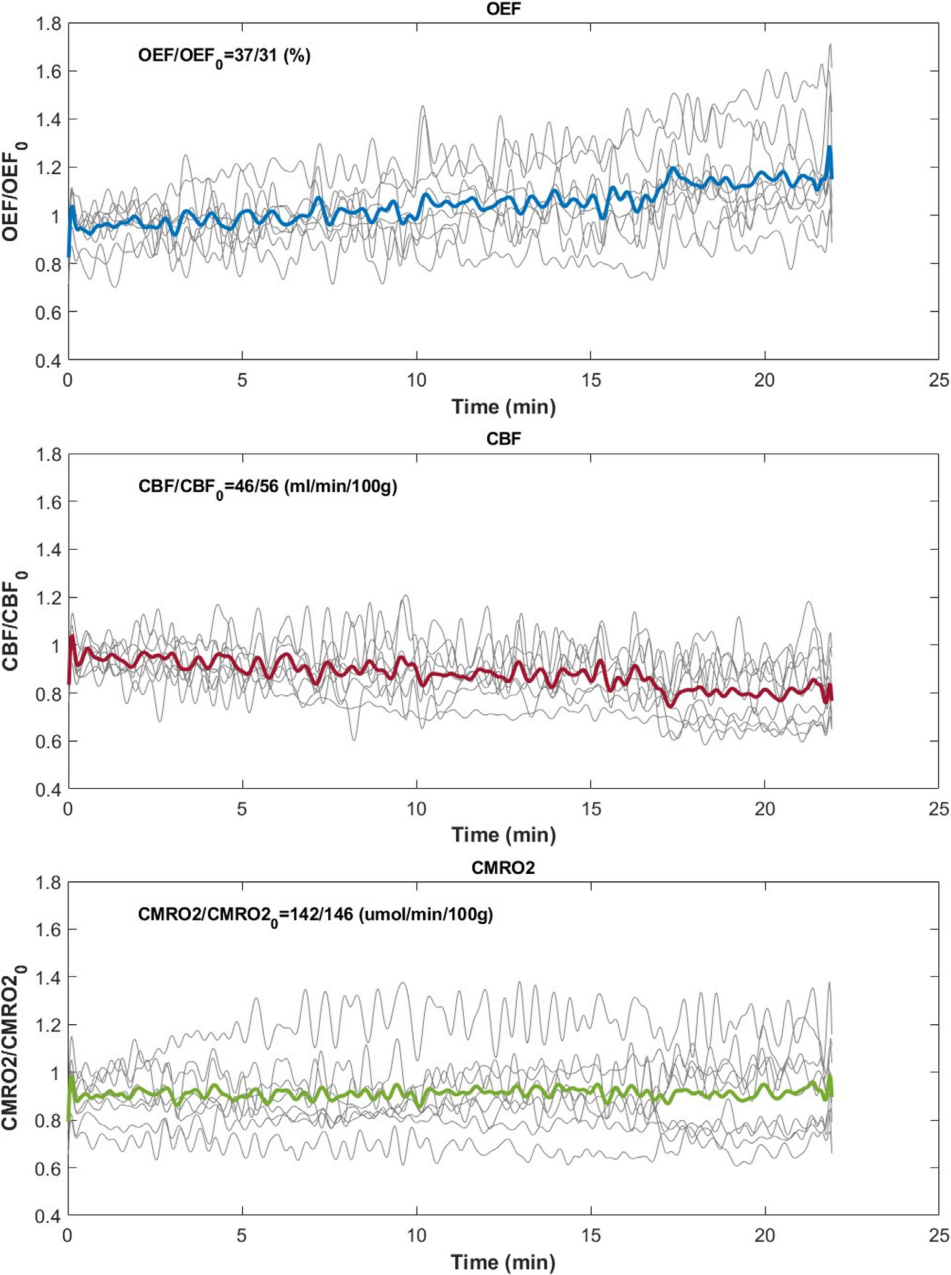
Representative temporal data obtained with the SS‐Oxflow sequence, covering a 30‐min time course obtained following administration of 200 mg of caffeine for OEF, CBF, and CMRO_2_ as indicated. Colored thick lines are means across the data from 10 subjects (thin lines). OEF shows a gradual increase over time, while CBF decreases and CMRO_2_ is maintained. Note substantial variability among subjects. The temporal data only encompasses ~22 min due to the time needed for set‐up and localization.

Figure 5 displays pre‐ and post‐caffeine OEF, CBF, and CMRO_2_ maps from two representative subjects, ages 33 (F) and 24 (M). There is a visually apparent decrease in CBF in both subjects, which is paired with a clear increase in OEF. In both subjects, there is no apparent change in CMRO_2_ pre versus post stimulus. Figure 6 depicts the time‐course data from all subjects. The mean temporal data demonstrated a gradual decrease in blood flow paired with an increase in OEF, which led to maintained CMRO_2_ while the caffeine was taking effect. However, these trends were variable across subjects, with some experiencing large changes in brain oxygen metabolism while a few subjects showed little to no change in metabolic parameters. Furthermore, CMRO_2_ was not invariant for several subjects.

**FIGURE 7:**
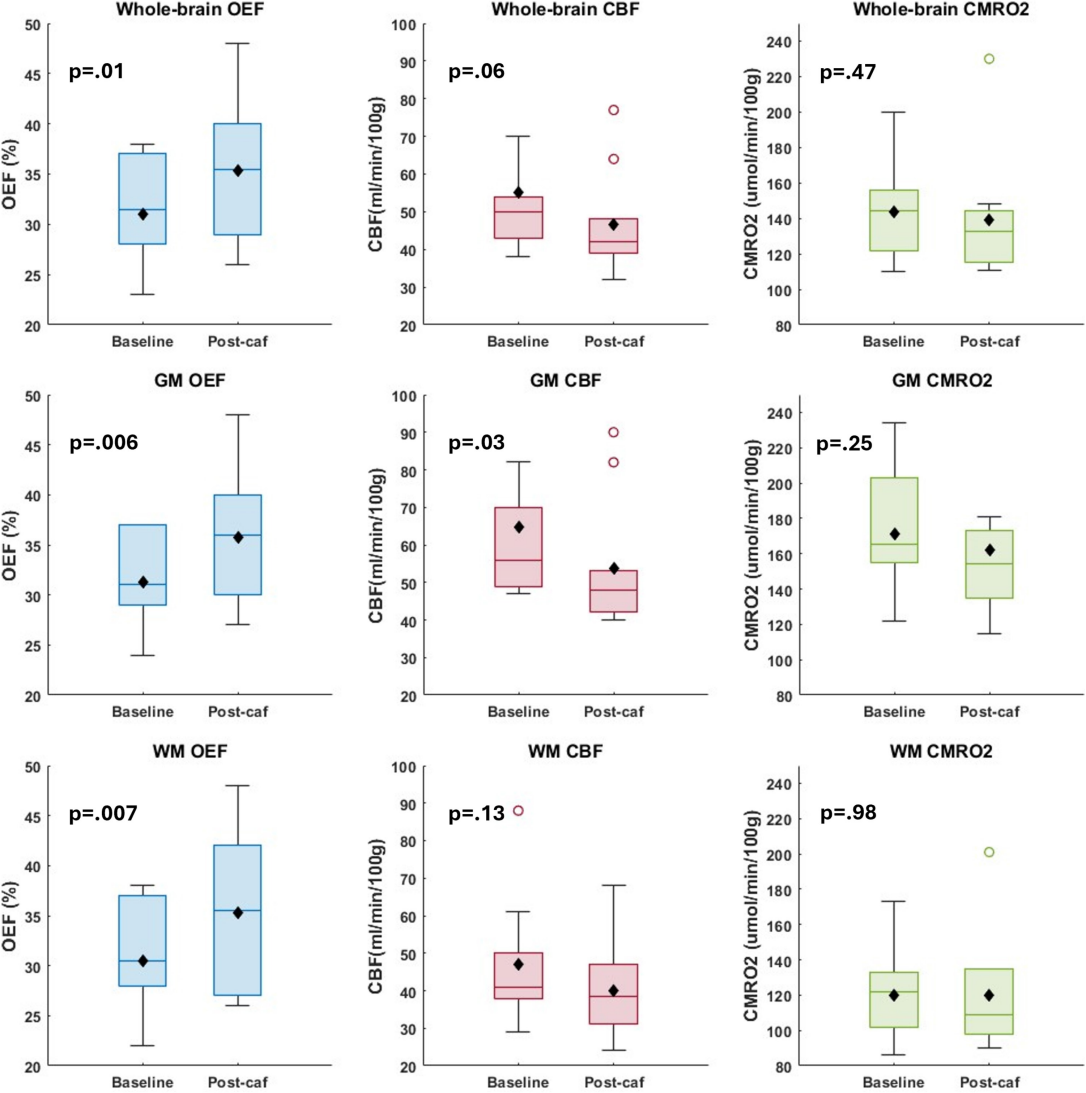
Global and regional effects of caffeine stimulus obtained with qBOLD as indicated. Whole‐brain averages as well as gray matter and white matter regions support the hypothesized response of vasoconstriction via caffeine challenge, i.e., reduced CBF and elevated OEF but no change in CMRO_2_.

A paired *t*‐test was performed for each technique, comparing pre‐ and post‐caffeine OEF, CBF, and CMRO_2_. Statistics from each ANOVA analysis are given in Table 1 for global techniques and whole‐brain averaged qBOLD, and in Table 3 for regional analysis. For all methods, CBF was found to be lower after caffeine consumption, while OEF was greater. However, CMRO_2_ did not differ between states (Figures S2 and 7). Regional analysis found OEF to differ significantly pre‐ and post‐intervention for both gray and WM and all other segmented brain regions, except the precentral gyrus. Further, CBF differed only significantly for GM. Figure 7 displays boxplots for whole‐brain, GM, and WM OEF, CBF, and CMRO_2_ pre‐ and post‐caffeine. The larger difference and variation in CBF pre‐ and post‐caffeine for GM produced a broader range of CMRO_2_ values compared with whole‐brain and WM measurements.

**TABLE 3:** Regional vascular‐metabolic parameters, pre vs. post caffeine, obtained from qBOLD parametric maps in 10 healthy subjects.

	OEF (%)			CBF (mL/min/100 g)			CMRO_2_ (μmol/min/100 g)		
	Pre	Post	*p*‐value^a^	Pre	Post	*p*‐value	Pre	Post	*p*‐value^a^
Gray matter	31 ± 5	36 ± 7	**0.006**	65 ± 23	54 ± 18	**0.03**	171 ± 33	162 ± 44	0.25
White matter	31 ± 6	35 ± 8	**0.007**	47 ± 17	40 ± 12	0.13	120 ± 26	120 ± 33	0.98
Caudate nucleus	32 ± 6	38 ± 9	**0.002**	40 ± 15	35 ± 17	0.41	107 ± 27	113 ± 39	0.56
Putamen	32 ± 6	38 ± 8	**0.01**	45 ± 18	36 ± 12	0.08	120 ± 34	115 ± 23	0.76
Pallidum	31 ± 7	39 ± 9	**0.01**	38 ± 16	32 ± 10	0.1	101 ± 37	104 ± 27	0.82
Thalamus	28 ± 9	35 ± 11	**0.01**	48 ± 15	42 ± 14	0.14	110 ± 27	126 ± 54	0.19
Hippocampus	26 ± 5	31 ± 8	**0.004**	53 ± 21	45 ± 17	0.1	113 ± 36	119 ± 38	0.26
Precentral gyrus^b^	28 ± 4	29 ± 6	0.54	70 ± 25	59 ± 23	0.07	169 ± 46	144 ± 43	**0.003**

*Note:* Significant *p*‐values are shown in bold.

Abbreviations: CBF, cerebral blood flow; CMRO_2_, cerebral metabolic rate of oxygen consumption; OEF, oxygen extraction fraction.

^a^ *p*‐values from paired *t*‐tests (*α* = 0.05).

^b^ The precentral gyrus contains the motor cortex.

## 4 Discussion

The present study evaluated the performance of the recently developed 3D constrained qBOLD method in comparison to some global (i.e., whole organ) oximetry methods. A second objective was to assess qBOLD's sensitivity to a vasoconstriction stimulus, both globally and regionally, in view of the method's potential for future clinical use.

Both at baseline and post‐caffeine, the results indicated that there was no significant bias between methods for OEF, the key parameter of interest (Figure 4). Also, qBOLD correlated more strongly with OxFlow methods than with MOTIVE. In addition, OxFlow methods had lower bias based on the Bland–Altman analysis. This observation can be understood based on the mechanistic similarities of OxFlow and qBOLD, which both rely on quantification of relative difference in magnetic susceptibility. In contrast, MOTIVE derives S_v_O_2_ by converting blood water T_2_, modulated by exchange/diffusion between chemical sites, via a calibration curve, analogous to TRUST.

The key determinant underlying the quantification of tissue S_v_O_2_ via qBOLD is the RF‐reversible transverse relaxation rate, R_2_′, which has contributions from both blood and non‐blood susceptibilities. Prior studies have shown that while T_2_‐based oximetric methods, such as TRUST, correlate well with susceptometry‐based methods [38], they have been found to yield lower values of S_v_O_2_, also relative to ground‐truth measurements obtained via venous puncture [41]. Similarly, the present study yielded S_v_O_2_ averages around 66% at baseline using MOTIVE compared with 68% for OxFlow and qBOLD techniques, and 60% post‐caffeine for MOTIVE compared with 62–63% for OxFlow and qBOLD. While small, these differences could adversely affect the strength of the correlation between MOTIVE and qBOLD. Additionally, the Bland–Altman plot in Figure 4 shows systemic positive bias, supporting the hypothesis that MOTIVE may yield higher OEF values.

Average baseline OEF across methods (31%) was slightly lower than reported in some PET studies [39, 47]. One reason for this may be the relatively small sample size (*N* = 10). In another study by Cho et al., comprising data from 10 healthy subjects, the mean value for OEF measured with ^15^O PET was 32.8 ± 6.7% [29]. Furthermore, the present study was conducted on a cohort of relatively young healthy subjects, most of whom were under the age of 35. Recent studies, such as the one by Jiang et al., found OEF to increase with age [48], and many PET studies of oxygen metabolism have been done on older as well as patient cohorts [47, 49, 50, 51]. On the other hand, strong agreement between methods at baseline (Table 1) leads to the conclusion that the measured OEF values are plausible.

In general, qBOLD yielded higher CMRO_2_ compared with global methods by about 10%–20% than the average from the three whole‐brain methods (Table 1). One reason for this is likely in the methods used for CBF quantification (pCASL versus PC). A prior, very large multi‐center study found a large bias in CBF between the two methods [52], yielding mean values of 55.8 for phase‐contrast and 47.7 for pCASL. That study reported a Pearson correlation coefficient of 0.59 between methods [52], similar to the present work, with an average correlation coefficient of 0.61 comparing pCASL (used for qBOLD derived CMRO_2_) and PC. However, Dolui et al. reported PC estimating higher values of CBF compared with ASL, while the present study (based on a much smaller sample of data with a different analysis method) showed the opposite. This mutual bias in CBF measurement between pCASL and PC‐derived CBF quantification is magnified for CMRO_2_ due to error propagation in Fick's principle equation (Equation 1, as CMRO_2_ is proportional to the product of OEF and CBF, which both have their own sources of error). Nevertheless, both CBF and CMRO_2_ changes relative to baseline were similar across all methods, regardless of the method used to measure blood flow (Table 1).

Caffeine stimulation resulted in a reduction in CBF with compensatory increases in OEF, found by qBOLD and the three whole‐brain methods, with no change in CMRO2, confirming the isometabolic nature of the stimulus, found for the majority of prior investigations [53, 54, 55] even though there was considerable spread in the magnitude of response among study subjects. Average fractional changes for OEF and CBF using qBOLD were comparable to those in previous studies [48, 54]. In distinction, Merola et al. reported caffeine stimulation to be non‐isometabolic, showing a decrease in CMRO_2_ [56]. Nevertheless, their data also showed a wide variation in caffeine response among subjects [56], similar to the present study.

Subregional analysis of the qBOLD data revealed significant differences in OEF response. GM and WM both showed significant increases in OEF after caffeine consumption. The precentral gyrus OEF was minimally affected. While not considered significant with *α* = 0.05, most regions also suggested reductions in CBF; though most of these trends did not reach significance, except for GM CBF, which indicated an average decrease of about 10 mL/min/100 g (*p* < 0.05). A significant increase in OEF in the basal ganglia supports previous fMRI results that there is more activation in this region post‐caffeine intake [57]. Overall, these findings support previous studies that the CBF response to caffeine stimulation differs across the brain [54, 57].

Clinical applications of the constrained qBOLD technique targeted include the study of cerebral oxygen metabolism in brain ischemia and injury. There has been some interest in evaluating the ischemic burden and iron load in patients with ischemic stroke, which has previously been investigated using QSM and relaxometric techniques [58, 59]. The presently used qBOLD technique, similar to QSM + qBOLD [13], enables separation of heme from non‐heme iron contributions in the brain [36], which could possibly enable a more accurate assessment of iron load in these patients. Finally, because qBOLD, in conjunction with ASL, yields CMRO_2_, additional insight into the ischemic burden of the affected tissue may be obtained, as shown in a study by Zhang et al. who demonstrated the ability of advanced qBOLD techniques to evaluate ischemic stroke patients [60].

There are several limitations of this study. First, the duration of the time‐resolved scans to observe the inflection point and return to baseline metabolic levels could not be achieved due to practical and ethical constraints. Furthermore, there was no blood or salivary measurement of caffeine concentration once the caffeine pill had been ingested, which may explain some of the disparities seen in response between subjects. With a relatively small sample size of 10 healthy participants, even 1–2 subjects with a dampened or heightened caffeine response could significantly influence group changes. In the future, validation by caffeine concentration measurement would be helpful to stratify results based on physiologically measured response. Moreover, not all scans were conducted at the same time of day, which may have played a role in response. Nevertheless, it is noted that, irrespective of the time of day, subjects were instructed to refrain from coffee consumption after 8:00 p.m. the night before, and no experiment was conducted before 8:00 a.m. or after 5:00 p.m. the next day. Additionally, while explicitly requested to stay awake during the scans, there was no verification of state of consciousness during the long procedure time, given that oxygen metabolism is altered during sleep [61], which could have impacted the results. Lastly, even though the magnitude and variance of the observed effects are commensurate with the number of subjects evaluated, studies involving larger sample sizes would be desirable. Finally, future work will include concatenation and acceleration of the two qBOLD sequences (AUSFIDE and VS‐VSL) as well as streamlining of the post‐processing pipeline to improve clinical utility and workflow.

## 5 Conclusion

The results suggest that constrained qBOLD yields brain vascular‐metabolic parameters comparable in magnitude to those from whole‐brain global MRI oximetry methods; further, the method has the sensitivity to detect the expected changes in oxygen metabolism in response to a vasoconstrictive stimulus, both globally and regionally.

## Conflicts of Interest

The authors declare no conflicts of interest.

## Supporting information


**Table S1:** Summary of global oximetry techniques.
**Table S2:** Summary of participant demographics.
**Figure S1:** Correlation matrices for (A) CBF and (B) CMRO_2_. SS‐OxFlow measurements correlate strongly with DS‐OxFlow.
**Figure S2:** Baseline vs. post‐caffeine for DS‐OxFlow (left column), SS‐OxFlow (middle column), and MOTIVE (right column). The first row is OEF, the second CBF, and the third CMRO_2_. OEF increased for all global techniques while CBF decreased, maintaining CMRO_2_.

## Data Availability

The data that supports the findings of this study are available in the Supporting Information of this article.

## References

[1] P. H. Wu , A. E. Rodriguez‐Soto , A. Wiemken , et al., “MRI Evaluation of Cerebral Metabolic Rate of Oxygen (CMRO_2_) in Obstructive Sleep Apnea,” Journal of Cerebral Blood Flow and Metabolism 42, no. 6 (2022): 1049–1060, 10.1177/0271678X211071018. 34994242 PMC9125486

[2] B. Cui , T. Zhang , Y. Ma , et al., “Simultaneous PET‐MRI Imaging of Cerebral Blood Flow and Glucose Metabolism in the Symptomatic Unilateral Internal Carotid Artery/Middle Cerebral Artery Steno‐Occlusive Disease,” European Journal of Nuclear Medicine and Molecular Imaging 47, no. 7 (2020): 1668–1677, 10.1007/s00259-019-04551-w. 31691843 PMC7248051

[3] N. Shen , S. Zhang , J. Cho , et al., “Application of Cluster Analysis of Time Evolution for Magnetic Resonance Imaging ‐Derived Oxygen Extraction Fraction Mapping: A Promising Strategy for the Genetic Profile Prediction and Grading of Glioma,” Frontiers in Neuroscience 15 (2021): 736891, 10.3389/fnins.2021.736891. 34671241 PMC8520989

[4] S. C. Cunnane , E. Trushina , C. Morland , et al., “Brain Energy Rescue: An Emerging Therapeutic Concept for Neurodegenerative Disorders of Ageing,” Nature Reviews. Drug Discovery 19, no. 9 (2020): 609–633, 10.1038/s41573-020-0072-x. 32709961 PMC7948516

[5] S. Dewanjee , P. Chakraborty , H. Bhattacharya , et al., “Altered Glucose Metabolism in Alzheimer's Disease: Role of Mitochondrial Dysfunction and Oxidative Stress,” Free Radical Biology & Medicine 193, no. Pt 1 (2022): 134–157, 10.1016/j.freeradbiomed.2022.09.032. 36206930

[6] A. P. Fan , H. An , F. Moradi , et al., “Quantification of Brain Oxygen Extraction and Metabolism With [^15^O]‐Gas PET: A Technical Review in the Era of PET/MRI,” NeuroImage 220 (2020): 117136, 10.1016/j.neuroimage.2020.117136. 32634594 PMC7592419

[7] W. Cao , Y. V. Chang , E. K. Englund , et al., “High‐Speed Whole‐Brain Oximetry by Golden‐Angle Radial MRI,” Magnetic Resonance in Medicine 79, no. 1 (2018): 217–223, 10.1002/mrm.26666. 28342212 PMC5612835

[8] R. S. Deshpande , M. C. Langham , C. C. Cheng , and F. W. Wehrli , “Metabolism of Oxygen via T_2_ and Interleaved Velocity Encoding: A Rapid Method to Quantify Whole‐Brain Cerebral Metabolic Rate of Oxygen,” Magnetic Resonance in Medicine 88, no. 3 (2022): 1229–1243, 10.1002/mrm.29299. 35699155 PMC9247043

[9] V. Jain , M. C. Langham , and F. W. Wehrli , “MRI Estimation of Global Brain Oxygen Consumption Rate,” Journal of Cerebral Blood Flow and Metabolism 30, no. 9 (2010): 1598–1607, 10.1038/jcbfm.2010.49. 20407465 PMC2949253

[10] F. Xu , Y. Ge , and H. Lu , “Noninvasive Quantification of Whole‐Brain Cerebral Metabolic Rate of Oxygen (CMRO_2_) by MRI,” Magnetic Resonance in Medicine 62, no. 1 (2009): 141–148, 10.1002/mrm.21994. 19353674 PMC2726987

[11] J. M. Oja , J. S. Gillen , R. A. Kauppinen , M. Kraut , and P. C. van Zijl , “Determination of Oxygen Extraction Ratios by Magnetic Resonance Imaging,” Journal of Cerebral Blood Flow and Metabolism 19, no. 12 (1999): 1289–1295, 10.1097/00004647-199912000-00001. 10598932

[12] G. A. Wright , B. S. Hu , and A. Macoviski , “Estimating Oxygen Saturation of Blood In Vivo With MR Imaging at 1.5 T,” Journal of Magnetic Resonance Imaging 1, no. 3 (1991): 275–283, 10.1002/jmri.1880010303. 1802140

[13] J. Cho , Y. Kee , P. Spincemaille , et al., “Cerebral Metabolic Rate of Oxygen (CMRO_2_) Mapping by Combining Quantitative Susceptibility Mapping (QSM) and Quantitative Blood Oxygenation Level‐Dependent Imaging (qBOLD),” Magnetic Resonance in Medicine 80, no. 4 (2018): 1595–1604, 10.1002/mrm.27135. 29516537 PMC6097883

[14] J. Cho , P. Spincemaille , T. D. Nguyen , A. Gupta , and Y. Wang , “Temporal Clustering, Tissue Composition, and Total Variation for Mapping Oxygen Extraction Fraction Using QSM and Quantitative BOLD,” Magnetic Resonance in Medicine 86, no. 5 (2021): 2635–2646, 10.1002/mrm.28875. 34110656 PMC10337202

[15] X. He and D. A. Yablonskiy , “Quantitative BOLD: Mapping of Human Cerebral Deoxygenated Blood Volume and Oxygen Extraction Fraction: Default State,” Magnetic Resonance in Medicine 57, no. 1 (2007): 115–126, 10.1002/mrm.21108. 17191227 PMC3971521

[16] H. An and W. Lin , “Quantitative Measurements of Cerebral Blood Oxygen Saturation Using Magnetic Resonance Imaging,” Journal of Cerebral Blood Flow and Metabolism 20, no. 8 (2000): 1225–1236, 10.1097/00004647-200008000-00008. 10950383 PMC4096835

[17] T. L. Davis , K. K. Kwong , R. M. Weisskoff , and B. R. Rosen , “Calibrated Functional MRI: Mapping the Dynamics of Oxidative Metabolism,” Proceedings of the National Academy of Sciences of the United States of America 95, no. 4 (1998): 1834–1839, 10.1073/pnas.95.4.1834. 9465103 PMC19199

[18] D. A. Yablonskiy , A. L. Sukstanskii , and X. He , “Blood Oxygenation Level‐Dependent (BOLD)‐Based Techniques for the Quantification of Brain Hemodynamic and Metabolic Properties—Theoretical Models and Experimental Approaches,” NMR in Biomedicine 26, no. 8 (2013): 963–986, 10.1002/nbm.2839. 22927123 PMC3510357

[19] H. Lu and Y. Ge , “Quantitative Evaluation of Oxygenation in Venous Vessels Using T2‐Relaxation‐Under‐Spin‐Tagging MRI,” Magnetic Resonance in Medicine 60, no. 2 (2008): 357–363, 10.1002/mrm.21627. 18666116 PMC2587050

[20] Z. B. Rodgers , V. Jain , E. K. Englund , M. C. Langham , and F. W. Wehrli , “High Temporal Resolution MRI Quantification of Global Cerebral Metabolic Rate of Oxygen Consumption in Response to Apneic Challenge,” Journal of Cerebral Blood Flow and Metabolism 33, no. 10 (2013): 1514–1522, 10.1038/jcbfm.2013.110. 23838827 PMC3790925

[21] P. A. Chiarelli , D. P. Bulte , R. Wise , D. Gallichan , and P. Jezzard , “A Calibration Method for Quantitative BOLD fMRI Based on Hyperoxia,” NeuroImage 37, no. 3 (2007): 808–820, 10.1016/j.neuroimage.2007.05.033. 17632016

[22] E. K. Englund , M. A. Fernandez‐Seara , A. E. Rodriguez‐Soto , et al., “Calibrated fMRI for Dynamic Mapping of CMRO_2_ Responses Using MR‐Based Measurements of Whole‐Brain Venous Oxygen Saturation,” Journal of Cerebral Blood Flow and Metabolism 40, no. 7 (2020): 1501–1516, 10.1177/0271678X19867276. 31394960 PMC7308517

[23] C. J. Gauthier and R. D. Hoge , “A Generalized Procedure for Calibrated MRI Incorporating Hyperoxia and Hypercapnia,” Human Brain Mapping 34, no. 5 (2013): 1053–1069, 10.1002/hbm.21495. 23015481 PMC6870118

[24] H. An and W. Lin , “Impact of Intravascular Signal on Quantitative Measures of Cerebral Oxygen Extraction and Blood Volume Under Normo‐ and Hypercapnic Conditions Using an Asymmetric Spin Echo Approach,” Magnetic Resonance in Medicine 50, no. 4 (2003): 708–716, 10.1002/mrm.10576. 14523956

[25] D. A. Yablonskiy and E. M. Haacke , “An MRI Method for Measuring *T* _2_ in the Presence of Static and RF Magnetic Field Inhomogeneities,” Magnetic Resonance in Medicine 37, no. 6 (1997): 872–876, 10.1002/mrm.1910370611. 9178238

[26] J. Ma and F. W. Wehrli , “Method for Image‐Based Measurement of the Reversible and Irreversible Contribution to the Transverse‐Relaxation Rate,” Journal of Magnetic Resonance. Series B 111, no. 1 (1996): 61–69, 10.1006/jmrb.1996.0060. 8620286

[27] H. Lee , E. K. Englund , and F. W. Wehrli , “Interleaved Quantitative BOLD: Combining Extravascular R_2_′ ‐ and Intravascular R_2_‐Measurements for Estimation of Deoxygenated Blood Volume and Hemoglobin Oxygen Saturation,” NeuroImage 174 (2018): 420–431, 10.1016/j.neuroimage.2018.03.043. 29580967 PMC5949279

[28] J. Zhang , T. Liu , A. Gupta , P. Spincemaille , T. D. Nguyen , and Y. Wang , “Quantitative Mapping of Cerebral Metabolic Rate of Oxygen (CMRO_2_) Using Quantitative Susceptibility Mapping (QSM),” Magnetic Resonance in Medicine 74, no. 4 (2015): 945–952, 10.1002/mrm.25463. 25263499 PMC4375095

[29] J. Cho , J. Lee , H. An , M. S. Goyal , Y. Su , and Y. Wang , “Cerebral Oxygen Extraction Fraction (OEF): Comparison of Challenge‐Free Gradient Echo QSM+qBOLD (QQ) With ^15^O PET in Healthy Adults,” Journal of Cerebral Blood Flow and Metabolism 41, no. 7 (2021): 1658–1668, 10.1177/0271678X20973951. 33243071 PMC8221765

[30] J. Cho , Y. Ma , P. Spincemaille , G. B. Pike , and Y. Wang , “Cerebral Oxygen Extraction Fraction: Comparison of Dual‐Gas Challenge Calibrated BOLD With CBF and Challenge‐Free Gradient Echo QSM+qBOLD,” Magnetic Resonance in Medicine 85, no. 2 (2021): 953–961, 10.1002/mrm.28447. 32783233 PMC7722021

[31] P. Elanghovan , T. Nguyen , P. Spincemaille , A. Gupta , Y. Wang , and J. Cho , “Sensitivity Assessment of QSM+qBOLD (or QQ) in Detecting Elevated Oxygen Extraction Fraction (OEF) in Physiological Change,” Journal of Cerebral Blood Flow and Metabolism 45, no. 4 (2025): 735–745, 10.1177/0271678X241298584. 39501700 PMC11951439

[32] J. Cho , S. Zhang , Y. Kee , et al., “Cluster Analysis of Time Evolution (CAT) for Quantitative Susceptibility Mapping (QSM) and Quantitative Blood Oxygen Level‐Dependent Magnitude (qBOLD)‐Based Oxygen Extraction Fraction (OEF) and Cerebral Metabolic Rate of Oxygen (CMRO_2_) Mapping,” Magnetic Resonance in Medicine 83, no. 3 (2020): 844–857, 10.1002/mrm.27967. 31502723 PMC6879790

[33] J. Cho , J. Zhang , P. Spincemaille , et al., “QQ‐NET—Using Deep Learning to Solve Quantitative Susceptibility Mapping and Quantitative Blood Oxygen Level Dependent Magnitude (QSM+qBOLD or QQ) Based Oxygen Extraction Fraction (OEF) Mapping,” Magnetic Resonance in Medicine 87, no. 3 (2022): 1583–1594, 10.1002/mrm.29057. 34719059 PMC9133659

[34] H. Lee and F. W. Wehrli , “Venous Cerebral Blood Volume Mapping in the Whole Brain Using Venous‐Spin‐Labeled 3D Turbo Spin Echo,” Magnetic Resonance in Medicine 84, no. 4 (2020): 1991–2003, 10.1002/mrm.28262. 32243708

[35] H. Lee and F. W. Wehrli , “Alternating Unbalanced SSFP for 3D R_2_′ Mapping of the Human Brain,” Magnetic Resonance in Medicine 85, no. 5 (2021): 2391–2402, 10.1002/mrm.28637. 33331076

[36] H. Lee and F. W. Wehrli , “Whole‐Brain 3D Mapping of Oxygen Metabolism Using Constrained Quantitative BOLD,” NeuroImage 250 (2022): 118952, 10.1016/j.neuroimage.2022.118952. 35093519 PMC9007034

[37] H. Lee , J. Xu , M. A. Fernandez‐Seara , and F. W. Wehrli , “Validation of a New 3D Quantitative BOLD Based Cerebral Oxygen Extraction Mapping,” Journal of Cerebral Blood Flow and Metabolism 44 (2024): 1184–1198, 10.1177/0271678X231220332. 38289876 PMC11179617

[38] S. Barhoum , Z. B. Rodgers , M. Langham , J. F. Magland , C. Li , and F. W. Wehrli , “Comparison of MRI Methods for Measuring Whole‐Brain Venous Oxygen Saturation,” Magnetic Resonance in Medicine 73, no. 6 (2015): 2122–2128, 10.1002/mrm.25336. 24975122 PMC4717477

[39] D. D. S. Jiang , C. G. Franklin , M. O'Boyle , et al., “Validation of T_2_‐Based Oxygen Extraction Fraction Measurement With ^15^O Positron Emission Tomography,” Magnetic Resonance in Medicine 83, no. 1 (2020): 68–82, 10.1002/mrm.28410. 32643207 PMC9973312

[40] Y. Ma , H. Sun , J. Cho , E. L. Mazerolle , Y. Wang , and G. B. Pike , “Cerebral OEF Quantification: A Comparison Study Between Quantitative Susceptibility Mapping and Dual‐Gas Calibrated BOLD Imaging,” Magnetic Resonance in Medicine 83, no. 1 (2020): 68–82, 10.1002/mrm.27907. 31373088

[41] X. Miao , K. S. Nayak , and J. C. Wood , “In Vivo Validation of T2‐ and Susceptibility‐Based S_v_O_2_ Measurements With Jugular Vein Catheterization Under Hypoxia and Hypercapnia,” Magnetic Resonance in Medicine 82, no. 6 (2020): 2188–2198, 10.1002/mrm.27871. PMC6717011 31250481

[42] K. R. Thulborn , J. C. Waterton , P. M. Matthews , and G. K. Radda , “Oxygenation Dependence of the Transverse Relaxation Time of Water Protons in Whole Blood at High Field,” Biochimica et Biophysica Acta 714, no. 2 (1982): 265–270, 10.1016/0304-4165(82)90333-6. 6275909

[43] M. Vidorreta , Z. Wang , Y. V. Chang , D. A. Wolk , M. A. Fernandez‐Seara , and J. A. Detre , “Whole‐Brain Background‐Suppressed pCASL MRI With 1D‐Accelerated 3D RARE Stack‐of‐Spirals Readout,” PLoS ONE 12, no. 8 (2017): e0183762, 10.1371/journal.pone.0183762. 28837640 PMC5570334

[44] K. J. Friston , *Statistical Parametric Mapping: The Analysis of Functional Brain Images* (Academic Press, 2011).

[45] B. Fischl , “FreeSurfer,” NeuroImage 62, no. 2 (2012): 774–781, 10.1016/j.neuroimage.2012.01.021. 22248573 PMC3685476

[46] H. Lu , F. Xu , K. Grgac , P. Liu , Q. Qin , and P. van Zijl , “Calibration and Validation of TRUST MRI for the Estimation of Cerebral Blood Oxygenation,” Magnetic Resonance in Medicine 67, no. 1 (2012): 42–49, 10.1002/mrm.22970. 21590721 PMC3158970

[47] J. P. Coles , T. D. Fryer , P. G. Bradley , et al., “Intersubject Variability and Reproducibility of ^15^O PET Studies,” Journal of Cerebral Blood Flow and Metabolism 26, no. 1 (2006): 48–57, 10.1038/sj.jcbfm.9600179. 15988475

[48] D. Jiang , P. Liu , Z. Lin , et al., “MRI Assessment of Cerebral Oxygen Extraction Fraction in the Medial Temporal Lobe,” NeuroImage 266 (2023): 119829, 10.1016/j.neuroimage.2022.119829. 36565971 PMC9878351

[49] “2024 Alzheimer's Disease Facts and Figures,” 2024. 10.1002/alz.13809 PMC11095490 38689398

[50] K. Kudo , T. Liu , T. Murakami , et al., “Oxygen Extraction Fraction Measurement Using Quantitative Susceptibility Mapping: Comparison With Positron Emission Tomography,” Journal of Cerebral Blood Flow and Metabolism 36, no. 8 (2016): 1424–1433, 10.1177/0271678X15606713. 26661168 PMC4976745

[51] H. Yamauchi , H. Fukuyama , Y. Nagahama , et al., “Evidence of Misery Perfusion and Risk for Recurrent Stroke in Major Cerebral Arterial Occlusive Diseases From PET,” Journal of Neurology, Neurosurgery, and Psychiatry 61, no. 1 (1996): 18–25, 10.1136/jnnp.61.1.18. 8676151 PMC486449

[52] S. Dolui , Z. Wang , D. J. J. Wang , et al., “Comparison of Non‐Invasive MRI Measurements of Cerebral Blood Flow in a Large Multisite Cohort,” Journal of Cerebral Blood Flow and Metabolism 36, no. 7 (2016): 1244–1256, 10.1177/0271678X16646124. 27142868 PMC4929707

[53] S. Buch , Y. Ye , and E. M. Haacke , “Quantifying the Changes in Oxygen Extraction Fraction and Cerebral Activity Caused by Caffeine and Acetazolamide,” Journal of Cerebral Blood Flow and Metabolism 37, no. 3 (2017): 825–836, 10.1177/0271678X16641129. 27029391 PMC5363462

[54] F. Xu , P. Liu , J. J. Pekar , and H. Lu , “Does Acute Caffeine Ingestion Alter Brain Metabolism in Young Adults?,” NeuroImage 110 (2015): 39–47, 10.1016/j.neuroimage.2015.01.046. 25644657 PMC4380776

[55] J. E. Perthen , A. E. Lansing , J. Liau , T. T. Liu , and R. B. Buxton , “Caffeine‐Induced Uncoupling of Cerebral Blood Flow and Oxygen Metabolism: A Calibrated BOLD fMRI Study,” NeuroImage 40, no. 1 (2008): 237–247, 10.1016/j.neuroimage.2007.10.049. 18191583 PMC2716699

[56] A. Merola , M. A. Germuska , E. A. Warnert , et al., “Mapping the Pharmacological Modulation of Brain Oxygen Metabolism: The Effects of Caffeine on Absolute CMRO_2_ Measured Using Dual Calibrated fMRI,” NeuroImage 155 (2017): 331–343, 10.1016/j.neuroimage.2017.03.028. 28323164 PMC7613100

[57] C. A. Park , C. K. Kang , Y. D. Son , et al., “The Effects of Caffeine Ingestion on Cortical Areas: Functional Imaging Study,” Magnetic Resonance Imaging 32, no. 4 (2014): 366–371, 10.1016/j.mri.2013.12.018. 24512799

[58] Y. Uchida , H. Kan , H. Inoue , et al., “Penumbra Detection With Oxygen Extraction Fraction Using Magnetic Susceptibility in Patients With Acute Ischemic Stroke,” Frontiers in Neurology 13 (2022): 752450, 10.3389/fneur.2022.752450. 35222239 PMC8873150

[59] Y. Uchida , H. Kan , Y. Kano , et al., “Longitudinal Changes in Iron and Myelination Within Ischemic Lesions Associate With Neurological Outcomes: A Pilot Study,” Stroke 55, no. 4 (2024): 1041–1050, 10.1161/STROKEAHA.123.044606. 38269537

[60] S. Zhang , J. Cho , T. D. Nguyen , et al., “Initial Experience of Challenge‐Free MRI‐Based Oxygen Extraction Fraction Mapping of Ischemic Stroke at Various Stages: Comparison With Perfusion and Diffusion Mapping,” Frontiers in Neuroscience 14 (2020): 535441, 10.3389/fnins.2020.535441. 33041755 PMC7525031

[61] J. Xu , A. Wiemken , M. C. Langham , et al., “Sleep‐Stage‐Dependent Alterations in Cerebral Oxygen Metabolism Quantified by Magnetic Resonance,” Journal of Neuroscience Research 102, no. 3 (2024): e25313, 10.1002/jnr.25313. 38415989 PMC12123640

