# Supplementary material for: Comparison of Brain Oxygen Metabolic Parameters Between Constrained qBOLD and Whole‐Brain Oximetric Methods at Baseline and in Response to a Physiologic Stimulus
Source: NMR Biomed. 2025 Aug 11;38(9):e70120. doi: 10.1002/nbm.70120 (PMC12337088; doi:10.1002/nbm.70120)

**Supplementary Table:** Summary of global oximetry techniques

|  | **DS-OxFlow** | **SS-OxFlow** | **MOTIVE** |
| --- | --- | --- | --- |
| **Method type** | Susceptibility-based | Susceptibility-based | T_2_-based |
| **Acquisition scheme** | Dual slice (S_v_O_2_ at SSS, CBF at neck) | Single slice (S_v_O_2_ and CBF at SSS) | Dual slice (S_v_O_2_ at SSS, CBF at neck) |
| **Readout** | Cartesian | Spiral | EPI |
| **Temporal resolution (s)** | 12 | 2 | 18 |
| **FOV (mm^2^)** | 230x230 | 224x224 | 230x230 |

Abbreviations: S_v_O_2_, venous oxygen saturation; SSS, superior sagittal sinus; CBF, cerebral blood flow; EPI, echo-planar imaging

| **Parameter** | **Value** |
| --- | --- |
| **Number of Participants** | 10 |
| **Age (years)** | 30 ± 8 (24 to 52) |
| **Weight (kg)** | 76 ±17 (57 to 118) |
| **Body mass Index (kg/m^2^)** | 25 ± 6 (21 to 41) |
| **Hematocrit (%)** | 41 ± 5 (36 to 48) |

**Supplementary Table 2:** Summary of Participant Demographics.

**Supplementary Figure 1:** Correlation matrices for (A) CBF and (B) CMRO_2_. SS-OxFlow measurements correlate strongly with DS-OxFlow.

**
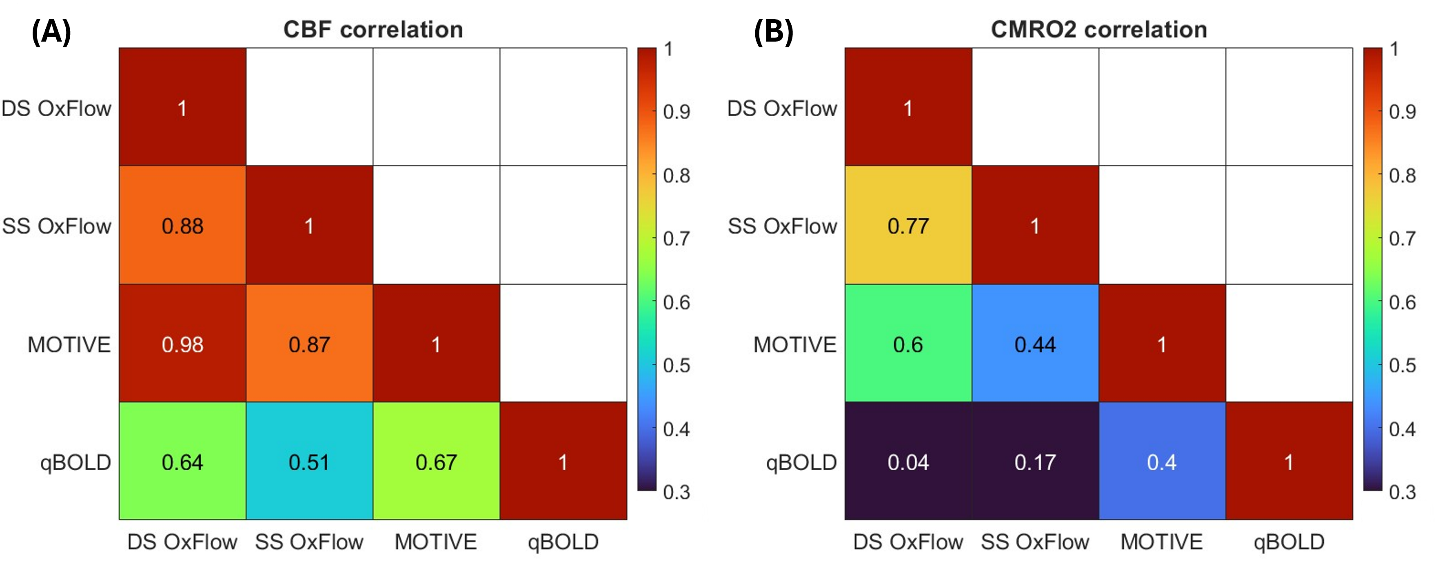
**

**Supplementary Figure 2:** Baseline vs. post-caffeine for DS-OxFlow (Left column), SS-OxFlow (Middle column), and MOTIVE (Right column). The first row is OEF, the second CBF, and the third CMRO_2_. OEF increased for all global techniques while CBF decreased, maintaining CMRO_2_.


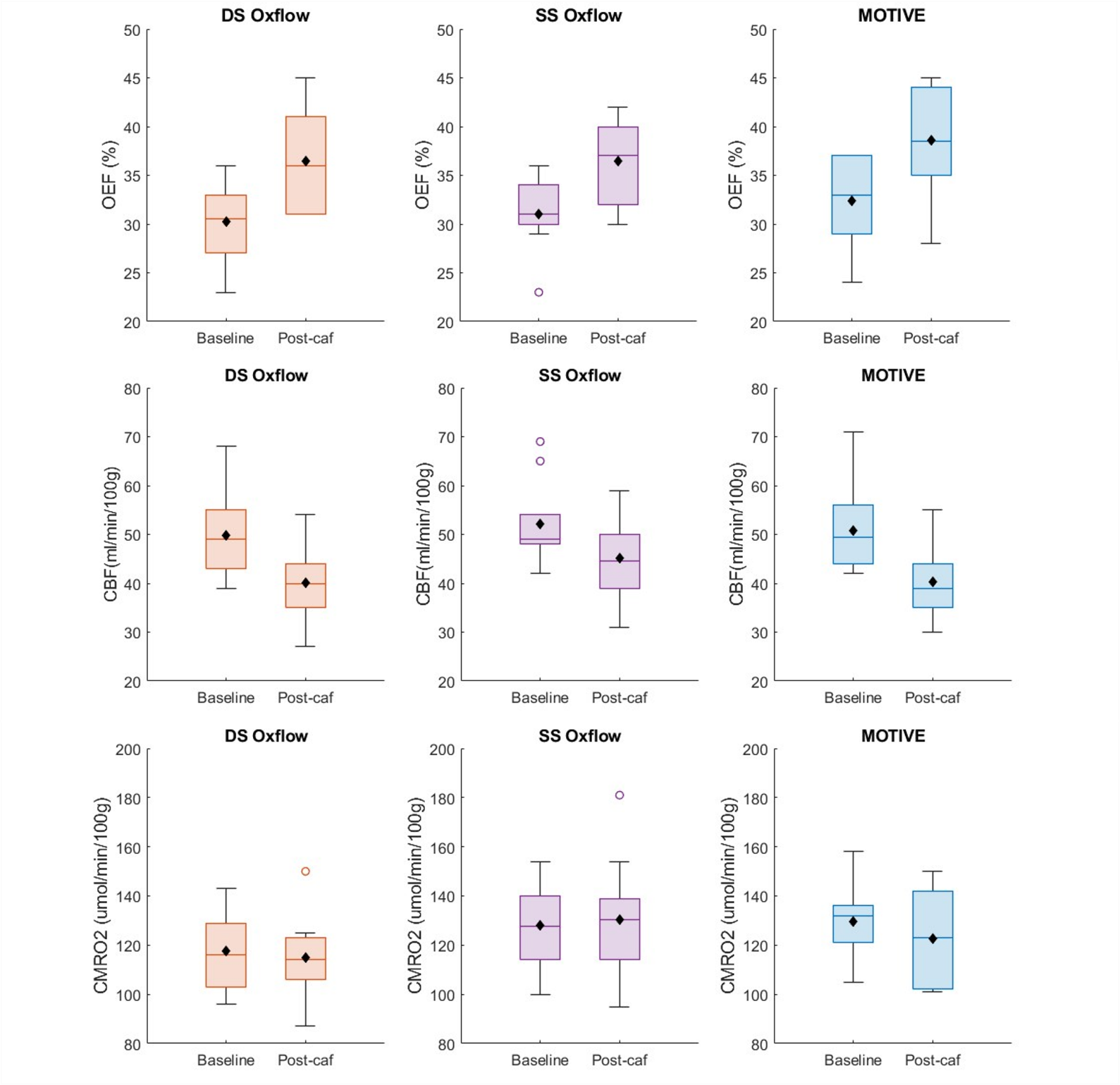

Supplement: Supplementary file 1 — Table S1: Summary of global oximetry techniques. Table S2: Summary of participant demographics. Figure S1: Correlation matrices for (A) CBF and (B) CMRO2. SS‐OxFlow measurements correlate strongly with DS‐OxFlow. Figure S2: Baseline vs. post‐caffeine for DS‐OxFlow (left column), SS‐OxFlow (middle column), and MOTIVE (right column). The first row is OEF, the second CBF, and the third CMRO2. OEF increased for all global techniques while CBF decreased, maintaining CMRO2. [file NBM-38-e70120-s001.docx]
